# Peripheral arterial disease and systematic detection of circulating tumor cells: rationale and design of the DETECTOR prospective cohort study

**DOI:** 10.1186/s12872-019-1193-1

**Published:** 2019-09-13

**Authors:** Alexandra Yannoutsos, Manon Fontaine, Alexandre Galloula, Diane Damotte, Gilles Chatellier, Patrizia Paterlini-Bréchot, Guy Meyer, Jean Pastre, Véronique Duchatelle, Valéria Marini, Karl-Léo Schwering, Isabelle Lazareth, Parinaz Ghaffari, Audrey Stansal, Hélène Sanson, Cécile Labrousse, Hélène Beaussier, Nesrine Ben Nasr, Marc Zins, Sergio Salmeron, Emmanuel Messas, Jean-Patrick Lajonchère, Joseph Emmerich, Pascal Priollet, Jean Trédaniel

**Affiliations:** 1 Groupe Hospitalier Paris Saint Joseph, 185 rue Raymond Losserand, 75 014 Paris, France; 2 Université Paris Descartes, Sorbonne Paris Cité, Paris, France; 3 Université Paris XIII, Villetaneuse, France; 4 Hôpital Européen Georges Pompidou, Assistance Publique – Hôpitaux de Paris, 75014 Paris, France; 5 Hôpital Cochin, Assistance Publique – Hôpitaux de Paris, 75014 Paris, France; 6 Unité INSERM UMR 1153-CRESS, Paris, France; 7 Unité INSERM U1138, Centre de Recherche des Cordeliers, Paris, France; 8 Unité INSERM UMR-S 1124, Toxicologie, pharmacologie et signalisation cellulaire, Paris, France

**Keywords:** Cancer, Tobacco, Screening, Circulating tumor cells

## Abstract

**Background:**

Smoking is a strong risk factor for cancer and atherosclerosis. Cancer mortality, especially from lung cancer, overtakes cardiovascular (CV) death rate in patients with peripheral arterial disease (PAD). Only a few patients with lung cancer after PAD management may benefit from surgical excision. Circulating tumor cells (CTC) associated with low-dose chest CT (LDCT) may improve early cancer detection. This study focuses on a screening strategy that can address not only lung cancer but all tobacco-related cancers in this high-risk population.

**Methods:**

DETECTOR Project is a prospective cohort study in two French University hospitals. Participants are smokers or former smokers (≥30 pack-years, quitted ≤15 years), aged ≥55 to 80 years, with atherosclerotic PAD or abdominal aortic aneurysm. After the first screening round combining LDCT and CTC search on a blood sample, two other screening rounds will be performed at one-year interval. Incidental lung nodule volume, volume doubling time and presence of CTC will be taken into consideration for adapted diagnostic management. In case of negative LDCT and presence of CTC, a contrast enhanced whole-body PET/CT will be performed for extra-pulmonary malignancy screening. Psychological impact of this screening strategy will be evaluated in population study using a qualitative methodology. Assuming 10% prevalence of smoking-associated cancer in the studied population, a total of at least 300 participants will be enrolled.

**Discussion:**

Epidemiological data underline an increase incidence in cancer and related death in the follow-up of patients with PAD, compared with the general population, particularly for tobacco-related cancers. The clinical benefit of a special workup for neoplasms in patients with PAD and a history of cigarette smoking has never been investigated. By considering CTCs detection in this very high-risk selected PAD population for tobacco-induced cancer, we expect to detect earlier pulmonary and extra-pulmonary malignancies, at a potentially curable stage.

**Trial registration:**

The study was registered in the French National Agency for Medicines and Health Products Safety (No N° EUDRACT_ID RCB: 2016-A00657–44) and was approved by the ethics Committee for Persons Protection (IRB number 1072 and n° initial agreement 2016-08-02; ClinicalTrials.gov identifier NCT02849041).

## Background

In European countries, cancer has now overtaken cardiovascular disease as the main cause of death [1]. Smoking is considered as a major risk factor for atherosclerosis and cancer and remains the predominant risk factor for premature death in Europe [2]. In patients with peripheral arterial disease (PAD), mortality rate from cancer is currently exceeding that of cardiovascular diseases [3]. Lung cancer is considered as the most frequent cause of death from cancer in Europe [4]. Most patients with tobacco related cancer present with advanced disease, not yet curable with currently available therapies. Therefore, early detection might be a valuable approach to detect the disease at an asymptomatic and potentially curable stage.

The clinical relevance of lung cancer screening in a population with PAD has been first underlined in 1983 in patients referred for peripheral vascular surgery [5]. Recent epidemiological evidence confirms that PAD as well as abdominal aortic aneurysm (AAA) are markers for the development of lung cancer [6–8], independently of age [9, 10]. Because of an advanced disease stage at the time of diagnosis, only a few patients with lung cancer after atherosclerotic vascular disease management may benefit from surgical excision [11]. Selection of patients with prevalent atherosclerotic peripheral disease may thus dramatically increase the benefit of cancer screening.

Early diagnosis of lung cancer is of paramount importance in terms of prognosis [12]. The US National Lung Screening Trial (NLST) pointed out that early lung cancer diagnosis has the potential to improve survival. A screening strategy with low-dose chest CT (LDCT) showed a 20% reduction in lung cancer-related deaths and an overall all-cause mortality reduction of 6,7% in participants with a history of cigarette smoking [13]. Results of the European NELSON study which proposed a screening protocol based on nodule volume measurement and growth rate confirms NLST results (as recently presented at the 2018 World Conference on Lung Cancer showing a reduction of lung cancer deaths by 26% in men and up to 39% in women).

However, LDCT screening focuses only on lung cancer, ignoring all other smoking related cancers (head and neck, esophagus, stomach, liver, colon, pancreas, kidney, bladder, ovary, uterine cervix and myeloid leukemia) [14]. On the other hand, strong concerns have been raised about possible harmful consequences of screening: false positive results requiring repeated and potential invasive procedures among participants without cancer, theoretical risk of radiation – induced carcinoma, and a poorly known psychological impact of screening procedures [15]. Screening should thus be applied to a population at high risk of smoking induced cancer.

Presence of circulating tumor cells (CTC) is considered as a sensitive biomarker for early metastatic cascade, cancer progression and drug treatment monitoring [16]. The Isolation by Size of Epithelial Tumor cells (ISET) technology is a blood filtration-based approach that has the potential to detect circulating non-hematologic cells [17]. Clinical data highlighted that these cells can be isolated in the blood stream before cancer was detectable on imaging [18].

In order to address not only lung cancer but all tobacco-related cancers in smokers and ex-smokers suffering from atherosclerotic PAD and/or AAA, the DETECTOR study will analyze the interest of CTC detection associated with LDCT for an early detection of cancers associated with PAD. The psychological part of the study will focus on emotional and psychosocial impact on participants of this screening strategy, and particularly those psychosocial mechanisms involved in the search of CTC. The psychological research team will also assess physician communication strategies.

## Methods/design

### Study design

DETECTOR project is a multicentric prospective cohort study, funded by the French National Cancer Institute, the National League against Cancer and the Foundation for Cancer Research, in a collaborative effort of Paris Descartes University associated hospitals and Paris Diderot University. This trial intends to evaluate unenhanced LDCT in combination with the detection of CTC, in the screening of smoking-associated cancers in patients with PAD or AAA as well as the psychological impact of this screening strategy.

After inclusion in the trial, participants will undergo the first round of screening. Participants without prevalent cancer at baseline will undergo two other screening rounds at one-year interval each, combining LDCT and CTC search on a blood sample (Table 1). Participants will be followed for a total of at least 3 years after inclusion.

**Table 1:** Study flow chart of examinations

Actions	V0: baseline visit	within 1 m after V0	V0 + 5w	V1(V0 + 1y)	V1 + 5w	V2(V0 + 2y)	V2 + 5w
Information	✓						
Informed consent	✓						
Verification of inclusion and exclusion criteria	✓						
Enrolment	✓						
Psychologist presence^(1)^	✓						
Personal and family medical history^(2)^	✓						
Ongoing treatments	✓			✓		✓	
Socio-demography	✓						
Level of tobacco use	✓						
Screening round: - Blood sampling for CTC detection - LDCT	✓			✓		✓	
Results of the screening round			✓		✓		✓
Psychological counselling^(3)^		✓	✓	✓			
Clinical events since last visit^(2)^				✓		✓	
Adverse events	✓		✓	✓	✓	✓	✓

1: as an observer; 2: focused on cancer and vascular diseases; 3: only for patients who are volunteer to participate at the psychological study

The study sponsor is Paris Saint-Joseph Hospital. The study was registered in the French National Agency for Medicines and Health Products Safety (No N° EUDRACT_ID RCB: 2016-A00657–44) and was approved by the ethics Committee for Persons Protection (Comité de Protection des Personnes Ile de France II, IRB number 1072 and n° initial agreement 2016-08-02). The total duration of the trial is 6 years (inclusion period = 36 months; follow-up = 3 years; ClinicalTrials.gov identifier NCT02849041). Prolonged follow-up of trial participants will be performed each year after the end of the scheduled trial period. The first study participant has been enrolled on January 2017; last patient’s enrollment is expected on December 2019.

### Study population

From January 2017, patients consulting for PAD or AAA in the departments of vascular medicine or surgery in Paris Saint-Joseph Hospital and European Georges Pompidou Hospital will be consecutively included after signed informed consent. Eligible participants, affiliated to the French social security system, are between 55 and 80 years of age at the time of inclusion, have a history of cigarette smoking of at least 30 pack-years, and, if former smokers, have quit within the previous 15 years. Active smoking patients at the time of inclusion in the study must engage in smoking cessation. Peripheral arterial disease is defined according to the following criteria: asymptomatic patients with ankle-brachial pressure index value less than 0.90 or pulse abolition with imaging-documented atherosclerotic vascular disease; symptomatic patients with intermittent claudication or chronic ischemic rest pain with or without tissue loss; acute limb ischemia; presence of AAA, defined as a localized dilation of the aorta of at least 50% in relation to the normal adjacent aorta measured by duplex-ultrasound or CT-scan.; previous arterial revascularization procedure of lower limb (by angioplasty or surgery) or amputation due to PAD.

Exclusion criteria are treatment for, or evidence of, any cancer within 5 years before enrollment, except non-melanoma skin cancer and in situ carcinomas, previous lung resection surgery, acute respiratory tract infection treated with antibiotics in the previous 12 weeks, contraindication to any invasive thoracic procedure, any sign suggestive of prevalent malignancy (unexplained weight loss ≥10% in the previous 12 months, recent hemoptysis…), renal insufficiency (creatinine clearance < 30 ml/mn) not allowing - if appropriate - injection of contrast medium, psychiatric comorbidities or limited life expectancy due to concomitant disease, ECOG Performance Status ≥2.

### Objectives

The primary objective of the study is to determine the diagnostic value of the couple LDCT combined with CTC search for the early detection of smoking associated cancers in a cohort of asymptomatic but high-risk participants presenting with PAD and/or AAA.

The secondary objectives are (1) to describe detected malignancies with this screening strategy and to evaluate patient’s survival, (2) to evaluate the rate of detection of CTCs in the study population, (3) to study the effect of smoking cessation program on patient’s decision to quit smoking, (4) to study the emotional and psychosocial impact of this screening strategy and more specifically related to CTC detection, (5) to assess, adjust and improve communication strategies of health professionals.

### Inclusion and screening procedures

Clear and detailed information is given to the patient before signed consent is obtained, emphasizing the annual nature of LDCT and blood tests and the possibility of abnormal results that may lead to additional investigations. If active smoker, he (she) is asked to consider smoking cessation and specific advice and supports are provided.

Enrolled participants undergo the first round of screening with LDCT and blood test sampling for CTCs detection. Multidetector scanner is performed with unenhanced acquisitions and low radiation exposure protocol consistent with lung cancer screening protocols [13, 19]. Each LDCT will result in an average effective dose ≤1.5 mSv. Search for CTCs with the ISET technique (Rarecells Diagnostics, Paris, France) is carried out as previously described [20]. Briefly, 10 mL of peripheral blood are collected in buffered EDTA and processed within 1 h of collection. The ISET method is a highly sensitive blood filtration-based approach using a polycarbonate membrane with pores of 8 μm. Most lymphocytes and neutrophils, whose diameter is between 8 and 10 μm, are not retained. Circulating cells, whose diameter is larger than 20 μm, are directly enriched with this technique and their morphology is conserved. Enriched cells are stained with cytological stainings (i.e., May Grunwald Giemsa) providing the possibility of analyzing the nuclear and cytoplasmic characteristics [21, 22]. Patients are considered positive for CTCs based on cytopathological malignant features determined by two experienced cytopathologists (DD, VD) according to previously defined criteria [18, 20, 22].

### Management of incidental findings

**Fig. 1:**
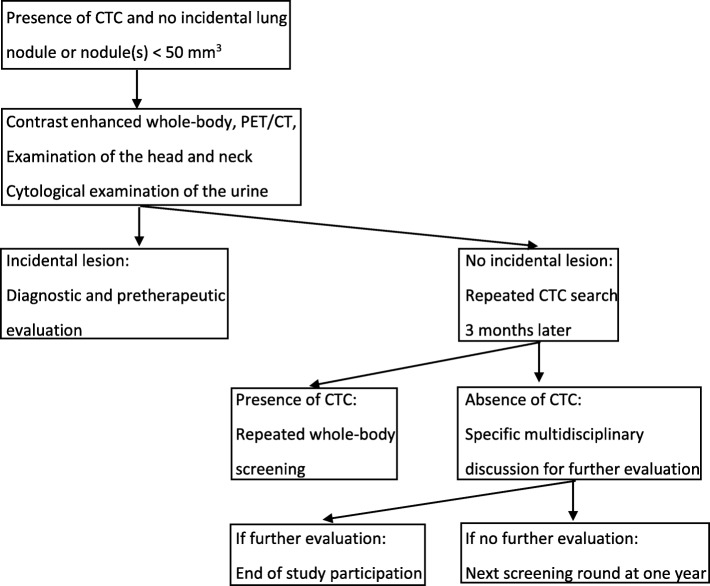
Adapted screening strategy in case of negative low-dose chest CT or incidental lung nodule(s) with a volume < 50 mm^3^ and presence of circulating tumor cells (CTCs)

First-line screening incidental findings and adapted screening strategy are displayed in Table 2. In case of negative LDCT or incidental lung nodule(s) with a volume < 50 mm^3^ and absence of CTCs, this screening strategy will be repeated once a year for a total of 3 consecutive rounds. Participants will be excluded from further screening when diagnosed with lung cancer or extra-pulmonary malignancy. In case of incidental lung nodule(s) with a volume > 500 mm^3^, in the presence or absence of CTCs, a specific multidisciplinary team meeting will discuss diagnostic and pre-therapeutic evaluation for lung cancer [23]. In case of incidental lung nodule(s) with a volume between 50 and 500 mm^3^ and presence of CTCs, diagnostic and pre-therapeutic evaluation for lung cancer will also be performed. In the absence of CTCs, volume doubling time - evaluated through a second LDCT performed three months later - will be taken into consideration for adapted diagnostic management. In case of negative LDCT or incidental lung nodule(s) with a volume < 50 mm^3^, and presence of CTCs, a contrast enhanced whole-body scanner and PET/CT associated with clinical head and neck examination and cytological examination of the urine will be performed for extra-pulmonary malignancy screening and management (Fig. 1).

**Table 2:** First-line screening incidental findings and adapted screening strategy

Low –dose chest CT and search for CTCs	No incidental lung nodule or nodule(s) <50mm^3^	Incidental lung nodule(s) with volume between 50 and 500mm^3^	Nodule(s) >500mm^3^
No CTC	Next screening round at one year	Measure of volume doubling time at low-dose chest CT 3 months later	Diagnostic and pretherapeutic evaluation
Presence of CTC	Contrast enhanced whole-body scanner and PET/CT examination of the head and neck cytological examination of the urine	Diagnostic and pretherapeutic evaluation	Diagnostic and pretherapeutic evaluation

*CTC: circulating tumor cells; PET/CT: Positron emission tomography-computed tomography*

### Collection of data

The usual demographic data will be recorded, including gender, age, weight, height, history of dyslipidemia, diabetes, hypertension, smoking habits, previous diseases and use of medications. History of coronary heart disease and cerebro-vascular disease will be recorded. For PAD, clinical and hemodynamic evaluations (ankle-brachial or toe-brachial index) will provide insights on the ischemia severity. All clinical, biological and imaging data will be anonymized and stored in electronic format.

### Statistical analysis and sample size

Data will be analyzed using SAS and R software. Alpha level for statistical significance (p) will be set at less than 5%. Characteristics of the study population will be described as mean plus or minus standard deviation for continuous variables with a normal distribution and as median [interquartile range] for skewness distribution. Categorical variables will be described as numbers and proportions. Student’s t-test or nonparametric Wilcoxon test will be used to compare quantitative parameters between different groups of patients and Chi2 test or Fischer test for qualitative parameters.

The primary outcome will focus on the sensibility and specificity of the screening strategy associating detection of CTCs and LDCT. Statistical analysis is in accordance with the STARD (Standards for Reporting of Diagnostic Accuracy Studies) guidelines [24]. To meet the primary objective, sensitivity will be estimated by dividing the number of patients diagnosed by the CTC method by the number of patients with lung cancer confirmed by the reference technique, i.e. LDCT. The results will be expressed with a binomial proportion 95% confidence interval. Specificity will be estimated by dividing the number of patients diagnosed with CTC as cancer-free with the number of patients without lung cancer. Secondary evaluation criteria will be analyzed using the same statistical methods as those used for the primary endpoint.

Assuming 10% prevalence of smoking-associated cancer in the studied population [8], if we aim to study a total of at least 30 detected cancers, including baseline and repeat screening rounds, we need to enroll a total of at least 300 participants.

### Psychological study

Volunteer patients will attend 3 individual interviews with the psychologist. The interviews will take place at key-moments of the procedure that are likely to trigger a significant emotional response: (1) within a month following the inclusion, (2) after the results of the first screening round, and (3) one year after the first screening round. The psychological study relies on the principles of the grounded theory approach [25]. It is inductive and iterative. The patient-physician communication will be quantitatively and qualitatively analyzed.

Individual interviews will be conducted with each of the volunteers. The psychologist has an active-listening role, asking clarification questions while enabling the patient to address other topics of his choice. Data collection will continue until theoretical saturation is achieved [26]. The psychological interviews will be qualitatively analyzed by two independent psychologists. Each psychologist will identify key points of the data (codes) within several levels of themes (categories). Data will be entered in N’Vivo software to facilitate the in-depth analysis of the material. Connections within and between categories will be identified until a meaningful theory is generated [27].

## Discussion

The originality of this study in a PAD / AAA population is to involve together, for the screening of cancer in high risk patients, clinicians, radiologists, pathologists as well as psychologists.

Lung cancer is the leading cause of cancer-related death worldwide [28]. This tumor is diagnosed in most cases at an advanced stage with prevalent distant metastases and reduced survival. By considering CTCs detection in a very high-risk selected population for tobacco-induced cancer, we expect to reduce false positive results of conventional imaging and to detect earlier pulmonary but also extra-pulmonary, malignancies when they are still curable.

Smoking cessation is an important factor in reducing mortality. In the NLST study, a cessation of smoking for at least 15 years associated with LDCT screening resulted in a 38% reduction in mortality, much higher than that achieved by the single effect of CT [29]. Participation in a screening study therefore presupposes for active smokers the parallel commitment in a smoking cessation procedure.

Cancer screening procedures have been associated to psychological adverse effects, particularly consequently to false positive results, over-diagnosis and over-treatment. Lung cancer screening causes an increase in anxiety [30], raising concerns about the short and long-term effects of screening on the psychological well-being of patients. By assessing the emotional and psychological impact of screening for cancers due to tobacco, we expect to identify 1/ specific fear, anxiety and psychological distress mechanisms, 2/ coping strategies that may benefit other patients, 3/ stress factors specifically linked to the screening procedure and/or to the medical information delivered to patients. By assessing the patient-doctor communication strategies, we expect to adjust and improve health professional communication skills respectful of patient psychological well-being.

Repetition of screening examinations raises the question of the risks associated with low-dose but repeated diagnostic irradiations. Irradiation delivered by a LDCT or a PET scanner is not negligible (1.5 mSv for LDCT) and may cause an excess risk of cancer. However, this risk appears much lower than the number of spontaneous cancers observed in a population at risk, which justifies the completion of these examinations [31]. In the NLST study, a positive examination was defined by the presence of at least one non-calcified nodule larger than or equal to 4 mm which led to a 27% positive test rate, 96% of which were false positives. We chose to apply the criteria of the NELSON study which demonstrated a significant reduction of false positive rate.

Common risk factors and pathophysiological processes have been highlighted in cancer and atherosclerosis, involving genetic alterations, inflammatory pathways, uncontrolled cell proliferation and pro-thrombotic processes [32]. Secondary cardiovascular prevention measures help delay vascular related events in PAD patients whereas epidemiological data underline an increase incidence in cancer and related death in the follow-up, compared with the general population, particularly for tobacco-related cancers [33, 34]. Large data from population-based cohort studies highlighted a 40% increased risk for cancer among patients with intermittent claudication [34, 35]. Prospective data reported a prevalence of 11.5% for malignancies in patients with critical leg ischemia and half of this population had died within 6 months [8]. However, not all of the patients with critical leg ischemia were investigated for occult malignancy, but only those with suggestive symptoms. The true prevalence of occult cancer in this population could thus be higher. The clinical benefit of a special workup for neoplasms in patients with PAD and a history of cigarette smoking has never been investigated. Furthermore, most patients diagnosed with cancer in the follow-up after PAD treatment exhibited advanced stage and poor survival [11, 34]. The clinical benefit of early cancer detection in this population may thus be expected. Although the lung is particularly susceptible for tobacco-induced cancer, a screening strategy should also have the potential to detect early stage neoplasm in all target organs. Circulating tumor cell search in the peripheral blood may represent a reliable tool suggestive for occult cancer in high risk PAD patients [18].

Different CTC isolation/detection methods exist. Transition of epithelial differentiation to mesenchymal phenotype is an important biological process leading to the generation of more aggressive sub-populations of CTCs. These invasive CTCs may not be captured with isolation/detection methods considering epithelial-lineage or tissue markers leading to false negative results, especially in patients with lung cancer [36]. The DETECTOR study will be the first prospective study analyzing the interest of CTC search with ISET technique in association with LDCT, possibly supplemented by whole-body imaging, in the screening strategy of smoking-associated cancers in PAD patients. A main challenge of the study is the detection method of CTCs, considering their low abundance in peripheral blood, fragility, heterogeneity and the lack of organ-specific markers [37]. If positive this study could pave the way for other cardiovascular population with a history of smoking, such as patients with coronary artery disease.

## Data Availability

Not applicable.

## Abbreviations

AAA: Abdominal aortic aneurysm
CT: Computed tomography
CTC: Circulating tumor cells
ECOG: Eastern cooperative oncology group
EDTA: Ethylene-diamine-tetraacetic acid
ISET: Isolation by size of epithelial tumor cells
LDCT: Low-dose chest CT
PAD: Peripheral arterial disease
PET/CT: Positron emission tomography–computed tomography
